# A Probiotic Mixture Neuralli™-CORE Attenuates DSS-Induced Colitis by Enhancing Gut Microbiota Resilience in Mice

**DOI:** 10.3390/ijms27115108

**Published:** 2026-06-04

**Authors:** Fu-Sheng Deng, Yu-Lin Cai, Wei-Hsiang Lin, Chien-Chen Wu, Ying-Chieh Tsai

**Affiliations:** 1 Research and Development Department, Bened Biomedical Co., Ltd., Taipei 115011, Taiwan; stephen@benedbiomed.com (F.-S.D.); yulincai@benedbiomed.com (Y.-L.C.); wei-hsiang.lin@benedbiomed.com (W.-H.L.); 2 Institute of Biochemistry and Molecular Biology, National Yang Ming Chiao Tung University, Taipei 11221, Taiwan

**Keywords:** DSS-induced colitis, multi-strain probiotic, gut microbiota, microbiota resilience, intestinal inflammation, microbial diversity, Neuralli^TM^-CORE

## Abstract

Maintenance of gut homeostasis is critical for overall health, as the gut microbiota plays a central role in regulating host metabolism, immune responses, and intestinal barrier integrity. Dysbiosis is closely associated with gastrointestinal disorders and inflammatory diseases, yet the ability of probiotics to preserve microbial resilience under inflammatory stress remains incompletely understood. In this study, we evaluated the protective effects of a multi-strain probiotic formulation, Neuralli^TM^-CORE (CORE), using a dextran sulfate sodium (DSS)-induced colitis mouse model. Mice were pre-supplemented with CORE for two weeks prior to DSS exposure. CORE supplementation significantly reduced disease activity index, increased body weight, and partially recovered the colonic histopathological damage in DSS-treated mice. Cytokine profiling showed that CORE reduced circulating PTX2, CHI3L1, CXCL13, and MMP-2 levels, suggesting attenuation of inflammation and tissue remodeling. Microbiota analysis revealed that CORE did not fully prevent DSS-induced dysbiosis but attenuated the early decline in α-diversity and promoted re-emergence of specific microbial taxa, including *Duncaniella* and *Muribaculum*, members of the *Muribaculaceae* family, which are inversely associated with inflammation. Correlation analysis further linked these taxa to reduced colitis severity. Collectively, CORE attenuates DSS-induced colitis by improving inflammatory resolution, supporting mucosal recovery, and enhancing microbiota resilience.

## 1. Introduction

In recent decades, rapid changes in dietary patterns, increasing environmental exposure, and heightened psychosocial stress have imposed significant challenges to intestinal health [1]. The gastrointestinal tract functions not only as a digestive organ but also as a critical interface for immune regulation and metabolic homeostasis [2]. Disruption of intestinal barrier integrity or perturbation of gut microbial communities can contribute to the development of inflammatory disorders, metabolic diseases, and immune dysregulation [3]. Moreover, accumulating evidence suggests that gut-derived pathological processes are associated with extraintestinal disorders, including neurological, hepatic, and cardiovascular diseases [4]. These findings highlight the importance of maintaining gut homeostasis as a central strategy for preserving overall health.

Probiotics are defined as live microorganisms that confer health benefits to the host when administered in adequate amounts [5]. Commonly, most probiotic species belong to the genera *Lactobacillus*, *Lactococcus*, and *Bifidobacterium*, many of which have been extensively investigated for their roles in maintaining intestinal homeostasis and regulating inflammatory responses [6]. Among them, *Bifidobacterium longum* BL21 has been reported to enhance epithelial barrier integrity, regulate mucosal immune responses, and reduce pro-inflammatory cytokine production in experimental colitis models [7]. Specific *Lacticaseibacillus rhamnosus* strains are known to reinforce tight junction function and modulate host immune signaling pathways associated with intestinal inflammation [8,9]. In addition, *Lacticaseibacillus paracasei* JYO062 has demonstrated anti-inflammatory and antioxidative properties through suppression of inflammatory mediators and modulation of gut immune responses [10]. *Limosilactobacillus fermentum* E7 has also been associated with antioxidant activity and regulation of reactive oxygen species and inflammatory cytokine production under intestinal inflammatory conditions [11]. Furthermore, *Lactococcus cremoris* PS133 has been implicated in the maintenance of mucosal homeostasis and host–microbiota interactions through the production of bioactive metabolites and modulation of intestinal immune function [12]. Based on these previous studies, these probiotic strains exhibit distinct biological activities associated with intestinal inflammation and gut microbial dysbiosis.

While individual strains may exert specific protective and immunomodulatory effects, gastrointestinal disorders are multifactorial and involve complex host-microbiota interactions. As such, single-strain interventions may provide incomplete coverage of these processes. Multi-strain probiotic formulations have therefore been proposed as a strategy to achieve broader modulation of gut microbial and immune functions [13]. For example, the combination of *L. rhamnosus* GG and *B. lactis* Bb12 has shown superior efficacy in pathogenic bacteria *Helicobacter pylori* eradication compared with either strain alone [14]. Furthermore, multi-strain probiotics have shown significantly greater efficacy in reducing the duration of diarrhea and hospitalization compared with single-strain probiotic *L. rhamnosus* GG [15]. Nevertheless, increasing formulation complexity may also introduce inter-strain competition or functional redundancy, potentially limiting overall efficacy [16]. These considerations highlight the need for rationally designed probiotic consortia that can effectively modulate gut homeostasis without compromising functional stability. Importantly, emerging evidence suggests that effective microbiota-targeted interventions may not necessarily restore microbial composition to a pre-disease state, but instead promote microbial resilience, the capacity of the microbial ecosystem to resist perturbation and recover following stress [17,18,19]. In the context of inflammatory conditions such as colitis, this may involve buffering disruption during the acute phase and facilitating re-establishment of a stable microbial configuration during recovery.

In this study, we evaluated the effects of a multi-strain probiotic formulation, Neuralli^TM^-CORE (CORE), in a dextran sulfate sodium (DSS)-induced colitis mouse model. Our results demonstrate that CORE supplementation attenuates DSS-induced colitis and modulates gut microbiota dynamics. Notably, CORE does not fully restore microbial diversity or community structure but instead enhances microbiota resilience, characterized by attenuation of early diversity loss during the acute phase and partial re-emergence of specific microbial features during recovery.

## 2. Results

### 2.1. CORE Attenuates DSS-Induced Colitis in Mice

**Figure 1:**
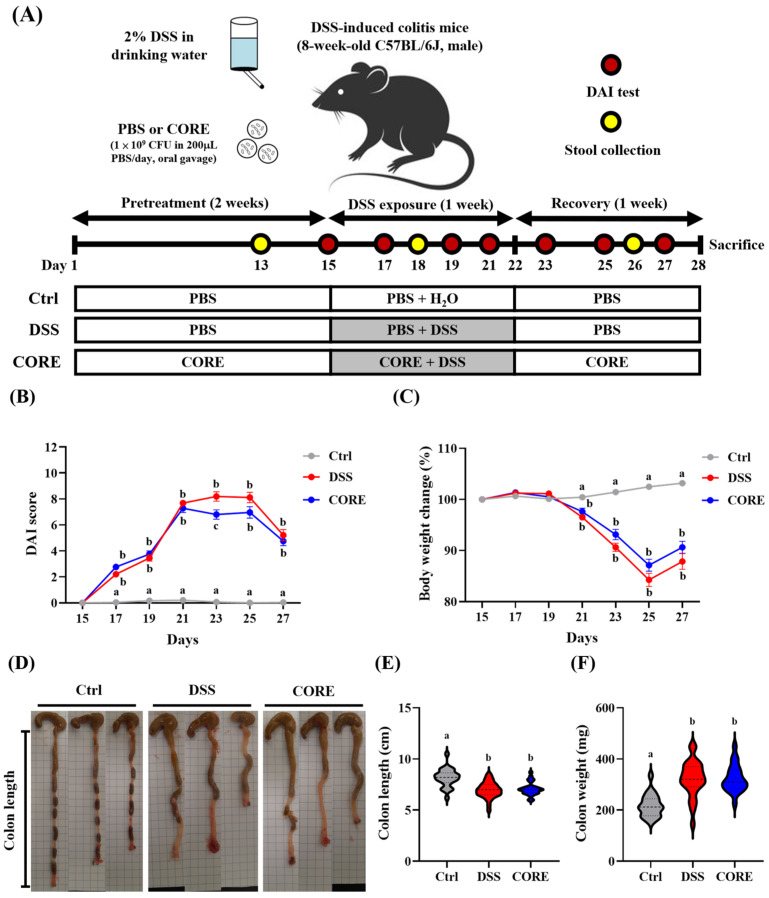
Effects of CORE supplementation on pathological indices in DSS-induced colitis mice. (**A**) Schematic illustration of the experimental design and treatment timeline. (**B**) Disease activity index (DAI) scores and (**C**) body weight changes across experimental groups. (**D**) Representative colon images from each group. Quantitation of (**E**) colon length and (**F**) colon weight. Data are presented as mean ± SEM from three independent experiments (*n* = 25–30 per group). Statistical significance was analyzed using one-way ANOVA followed by Tukey’s post hoc test. Different letters (a, b, c) indicate statistically significant differences between groups (*p* < 0.05).

To evaluate the protective effect of CORE supplementation against intestinal inflammation, we employed a DSS-induced colitis mouse model. The experimental design and grouping strategy are illustrated in Figure 1A. Mice were orally administered PBS or CORE (1 × 10^9^ CFU in 200 μL PBS daily), beginning on day 1 and continuing throughout the experimental period. Colitis was induced by administering 2% DSS in the drinking water for 7 days, followed by a 7-day recovery period with regular drinking water. Disease activity index (DAI) assessment and stool collection were performed according to the experimental timeline. As shown in Figure 1B, DAI scores began to rise from day 2 of DSS exposure (day 17) and reached a plateau by day 21. The elevated disease severity persisted after DSS withdrawal and gradually declined, with a significant reduction observed on day 27. Although comparable disease severity was observed between DSS-treated groups during the induction phase, CORE-fed mice exhibited a significantly faster recovery compared with DSS group (DAI score: 6.8 ± 0.37 vs. 8.2 ± 0.37 for CORE vs. DSS, respectively; *p* = 0.0055, day 23) (Figure 1B). Consistent with DAI changes, DSS exposure induced significant body weight loss, whereas CORE supplementation attenuated DSS-induced weight loss (Body weight change: 93.14 ± 5.03% vs. 90.64 ± 4.74% for CORE vs. DSS, respectively; *p* = 0.057, day 23) (Figure 1C).

DSS exposure is well known to induce colon shortening, a hallmark of colonic inflammation [20]. In our study, 7 days of DSS administration followed by a 7-day recovery period resulted in a significant reduction in colon length (6.96 ± 0.14 vs. 8.17 ± 0.18 cm, DSS vs. Ctrl, respectively; *p* < 0.0001) (Figure 1D,E) and a concomitant increase in colon weight (322.1 ± 14.11 vs. 218.3 ± 9.88 mg, DSS vs. Ctrl, respectively; *p* < 0.0001) (Figure 1F). Despite significantly reducing DAI scores, CORE supplementation did not restore colon length or weight to control levels (CORE: 7.09 ± 0.13 cm and 323.9 ± 11.93 mg; *p* > 0.05 vs. DSS) (Figure 1E,F). Collectively, these findings indicate that CORE improves disease activity but does not fully reverse DSS-induced colon shortening.

### 2.2. CORE Attenuates DSS-Induced Histopathological Alterations in the Colon

**Figure 2:**
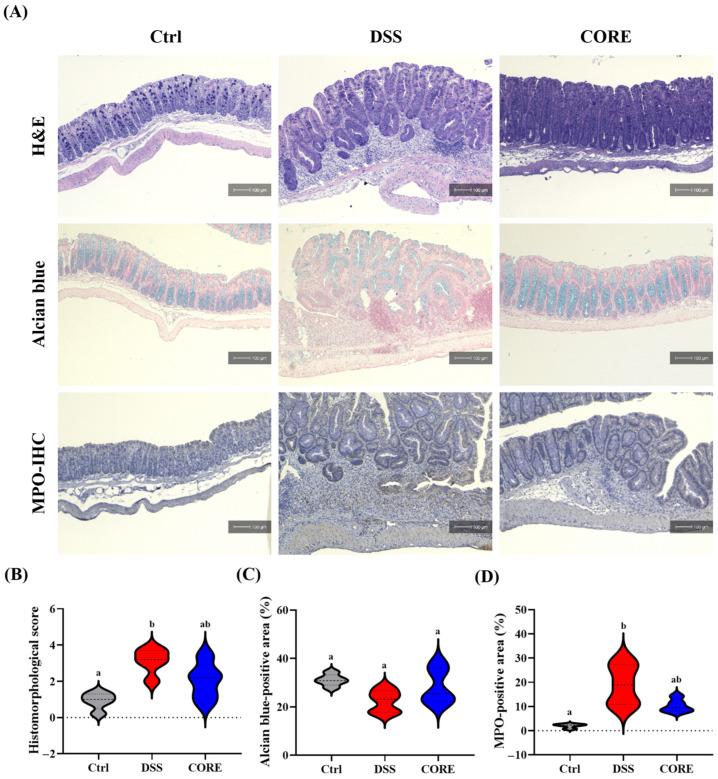
Effects of CORE on histopathological alterations in DSS-induced colitis. (**A**) Representative images of colonic tissues stained with hematoxylin and eosin (H&E), Alcian blue, and myeloperoxidase immunohistochemistry (MPO-IHC) to evaluate tissue morphology, goblet cell integrity, and neutrophil infiltration, respectively. Scale bar = 100 μm. (**B**) H&E histological scores, (**C**) Alcian blue-positive area, and (**D**) MPO-positive area. Data are presented as mean ± SEM (*n* = 5 per group). Statistical significance was analyzed using one-way ANOVA followed by Tukey’s post hoc test. Different letters (a, b) indicate statistically significant differences between groups (*p* < 0.05).

Given that DSS-induced colitis is associated with pronounced structural and inflammatory damage to the colonic mucosa, including epithelial erosion, crypt shortening, mucosal edema, and immune cell infiltration within the lamina propria [21], we next examined whether CORE supplementation could mitigate these tissue-level alterations. As shown in Figure 2A, DSS treatment induced marked epithelial disruption and crypt damage, which were reflected by significantly elevated histological scores compared with the Ctrl group (3.12 ± 0.31 vs. 0.92 ± 0.21, DSS vs. Ctrl, respectively; *p* = 0.002) (Figure 2B). CORE supplementation partially improved tissue morphology, with a trend toward reduced histological scores (CORE: 2.04 ± 0.44, *p* = 0.099 vs. DSS) (Figure 2B). To further assess the colonic mucus barrier, Alcian blue staining was performed. DSS-treated mice exhibited a substantial loss of goblet cells and reduced mucin-positive areas (22.39 ± 2.05% vs. 31.07 ± 1.09%, DSS vs. Ctrl, respectively; *p* = 0.053), whereas CORE supplementation led to a restoration of Alican blue-positive regions; however, no statistically significant difference was observed (CORE: 28.42 ± 3.30%, *p* > 0.05 vs. DSS) (Figure 2A,C). Myeloperoxidase (MPO), a neutrophil-derived enzyme and established marker of inflammatory infiltration [20], was assessed by immunohistochemistry (IHC). MPO-IHC analysis revealed pronounced neutrophil accumulation in the DSS group, whereas CORE supplementation reduced MPO-positive areas (10.29 ± 1.20 vs. 19.03 ± 3.73%, CORE vs. DSS, respectively; *p* = 0.059), indicating attenuation of mucosal inflammation (Figure 2A,D). Collectively, these histological and quantitative findings indicate a modest attenuation of DSS-induced colonic damage following CORE supplementation; however, the differences did not reach statistical significance.

### 2.3. CORE Attenuates DSS-Induced Inflammatory Responses

**Figure 3:**
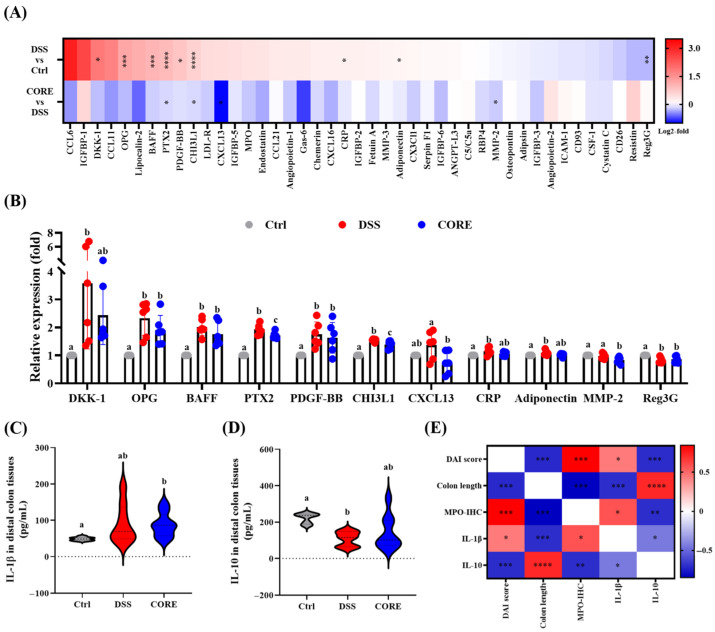
CORE attenuates colitis-associated inflammatory responses and protein expression in the distal colon. (**A**,**B**) Heatmap and quantitative analysis of cytokine expression profiles among Ctrl, DSS, and CORE groups based on serum cytokine array results. Data are representative of three independent experiments (*n* = 3 per group). (**C**,**D**) Quantitation of IL-1β and IL-10 levels in the distal colon, with statical significance analyzed using Welch’s ANOVA or ordinary ANOVA, respectively (*n* = 8 per group). (**E**) Heatmap of Spearman’s correlation analysis among intestinal physiological and pathological parameters. Different letters (a, b, c) indicate statistically significant differences between groups (*p* < 0.05). Color intensity indicates the strength and direction of the correlation (red, positive; blue, negative), and asterisks denote statistical significance. * *p* < 0.05, ** *p* < 0.01, *** *p* < 0.001, **** *p* < 0.0001.

To evaluate the effects of CORE on DSS-induced colonic inflammation, we profiled systemic inflammatory mediators in the serum using a cytokine array capable of detecting 111 mouse soluble proteins. As shown in Figure 3A, DSS administration markedly altered the circulating cytokine profile after the recovery phase (day 28), with eight factors, Dickkopf-1 (DKK-1), Osteoprotegerin (OPG), B-cell activating factor (BAFF), Pentraxin 2 (PTX2), Platelet-derived growth factor-BB (PDGF-BB), Chitinase 3-like 1 (CHI3L1), C-Reactive Protein (CRP), and adiponectin, significantly elevated, while regenerating islet-derived 3 gamma (Reg3G) was significantly reduced compared with the Ctrl group. This pattern suggests that, despite DSS withdrawal, mice remained in a state of ongoing post-inflammatory remodeling characterized by persistent low-grade inflammation, epithelial stress, and incomplete restoration of mucosal barrier function. Notably, CORE supplementation significantly reduced circulating PTX2 and CHI3L1 levels compared with the DSS group (Figure 3B). Given that CHI3L1 is associated with persistent mucosal inflammation and epithelial stress [22], and PTX2 reflects ongoing tissue injury and repair-related remodeling [23], these findings suggest that CORE may facilitate resolution of residual inflammation and promote more efficient mucosal recovery following DSS-induced colitis. In addition, CORE treatment also significantly reduced Chemokine C-X-C motif ligand 13 (CXCL13) and Matrix metalloproteinase-2 (MMP-2) levels, although neither marker was significantly altered by DSS alone. CXCL13 is linked to adaptive immune cell recruitment and chronic inflammatory signaling [24], whereas MMP-2 is involved in extracellular matrix turnover and tissue remodeling [25]. The selective reduction of these mediators by CORE may therefore indicate an active enhancement of mucosal resilience and structural restoration beyond spontaneous recovery. Together, these results support a role for CORE in alleviating DSS-induced systemic inflammatory disturbances and improving the quality of post-inflammatory healing.

Notably, cytokine alterations were not uniformly observed between serum and tissue [26]. While IL-1β and IL-10 levels were significantly altered in colonic tissue [27], these changes were not detected in the serum using the cytokine array. To further investigate this, cytokine expression was assessed in distal colon tissues. As shown in Figure 3C and 3D, DSS administration altered IL-1β and IL-10 levels in the distal colon (IL-1β: 91.0 ± 19.21 vs. 48.8 ± 1.73 pg/mL, *p* = 0.082; IL-10: 107.8 ± 14.22 vs. 221.5 ± 9.76 pg/mL, *p* = 0.0038; DSS vs. Ctrl, respectively). CORE supplementation partially attenuated these DSS-induced changes (IL-1β: 87.3 ± 12.1 pg/mL; IL-10: 145.4 ± 33.6 pg/mL, CORE vs. DSS, respectively). To explore the relationship between cytokine modulation and disease severity, Spearman’s correlation analysis was performed. Changes in IL-1β and IL-10 levels were significantly correlated with DAI, colon length, and MPO-IHC (Figure 3E). Collectively, these findings indicate that CORE supplementation is associated with reduced inflammatory cytokine levels in the serum and colonic tissue in the DSS model.

### 2.4. CORE Enhances Microbiota Resilience in DSS-Induced Colitis

**Figure 4:**
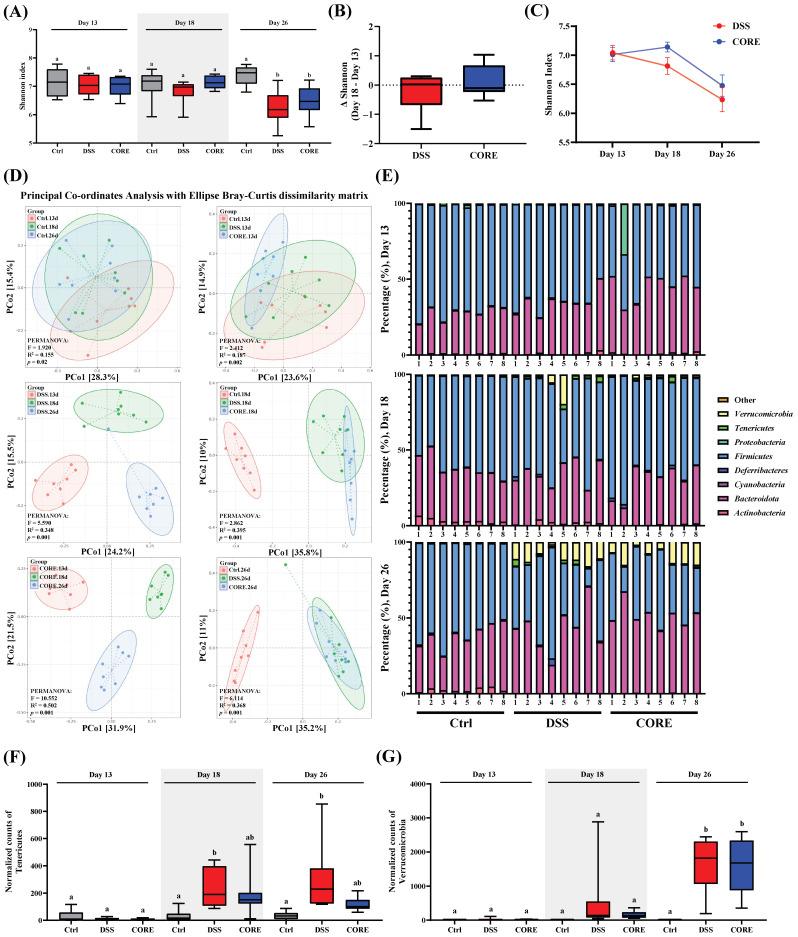
Effects of CORE on gut microbiota composition in DSS-induced colitis mice. (**A**) α-diversity assessed by the Shannon index. (**B**) Change in Shannon diversity index (ΔShannon) between day 13 and day 18 during DSS exposure period. (**C**) Temporal changes in Shannon diversity index across experimental groups. (**D**) β-diversity assessed by principal coordinates analysis (PCoA) based on distance metrics to visualize differences in microbial community structure. (**E**) Gut microbiota composition at the phylum level. Quantitative comparison of the relative abundance of (**F**) Tenericutes and (**G**) Verrucomicrobia across groups. Data are presented as mean ± SEM (*n* = 8 per group). Statistical significance was analyzed using one-way ANOVA or Welch’s *t*-test. Different letters (a, b) above the bars indicate statistically significant differences at *p* < 0.05.

Then, we performed gut microbiota profiling to determine whether CORE supple mentation could influence microbial diversity in DSS-induced colitis. Analysis of α-diversity using the Shannon index showed that mice in the Ctrl group maintained stable microbial diversity throughout the experimental period, with no significant temporal changes observed (days 13, 18, and 26; Figure 4A). Consistent results were obtained using Simpson and Pielou’s evenness indices (Figure S1). At baseline (day 13), no significant differences were detected among groups, confirming comparable microbiota composition prior to DSS exposure. In contrast, DSS-treated mice exhibited a progressive decline in microbial diversity, with a reduction evident on day 18 and a significant decrease on day 26 compared with the Control group, indicating sustained microbiota disruption following DSS exposure (Figure 4A). Notably, CORE supplementation attenuated this early decline, maintaining microbial diversity at levels comparable to baseline during the DSS challenge phase (day 18). To further quantify this effect, changes in Shannon diversity between day 13 and day 18 were calculated. DSS-treated mice exhibited a decrease in α-diversity (ΔShannon = −0.228), whereas CORE-treated mice showed no decline (ΔShannon = 0.128). Although this difference did not reach statistical significance (*p* = 0.249; Figure 4B), the opposing directional trends suggest that CORE attenuates DSS-induced loss of microbial diversity during the acute phase. Consistent with these findings, temporal trajectory analysis demonstrated that DSS-treated mice exhibited a continuous decline in Shannon diversity from day 13 to day 26, whereas CORE-treated mice maintained relatively stable diversity during DSS exposure, followed by a decline during the recovery phase (Figure 4C). No significant group or group × time interaction was observed; however, the overall pattern supports a buffering effect of CORE on microbiota disruption under inflammatory stress. Collectively, these findings indicate that CORE enhances microbiota resilience by mitigating DSS-induced disruption during the acute inflammatory phase, rather than promoting full restoration of microbial diversity after injury.

β-diversity analysis based on principal coordinates analysis (PCoA) demonstrated that DSS treatment markedly altered the overall microbial community structure compared with the Control group (Figure 4D). In contrast, the microbial profiles of DSS and CORE groups largely overlapped, indicating that CORE supplementation did not substantially restore DSS-induced shifts in β-diversity or global community composition. Phylum-level analysis compared the percentages of eight dominant bacterial phyla, including Actinobacteria, Bacteroidota, Cyanobacteria, Deferribacteres, Firmicutes, Proteobacteria, Tenericutes, and Verrucomicrobia (Figure 4E and Figure S2). Among the dominant phyla examined, Tenericutes drew particular attention due to its pronounced and dynamic alterations across both the induction and recovery phases. Specifically, during the recovery phase, Tenericutes remained elevated in DSS-treated mice, indicating persistent microbiota imbalance following DSS exposure. In contrast, CORE supplementation restored Tenericutes abundance to levels comparable to the Ctrl group (Figure 4F). In comparison, Verrucomicrobia exhibited an increasing trend during DSS exposure and was significantly elevated during the recovery phase in both DSS and CORE groups. However, we still observed a modestly lower level in CORE-treated mice on day 18 (Figure 4G). Given that Verrucomicrobia is largely represented by mucin-degrading bacteria such as *Akkermansia muciniphila* [28], this pattern may reflect DSS-induced alterations in the mucus layer, with CORE exerting only a limited effect on restoring mucosal niche structure. Collectively, these findings suggest that CORE promotes normalization of dysbiosis-associated taxa in a selective manner. Consistent with the β-diversity results, this effect does not reflect a global restructuring of the microbial community.

### 2.5. CORE-Associated Microbial Taxa Correlate with Disease Severity

**Figure 5:**
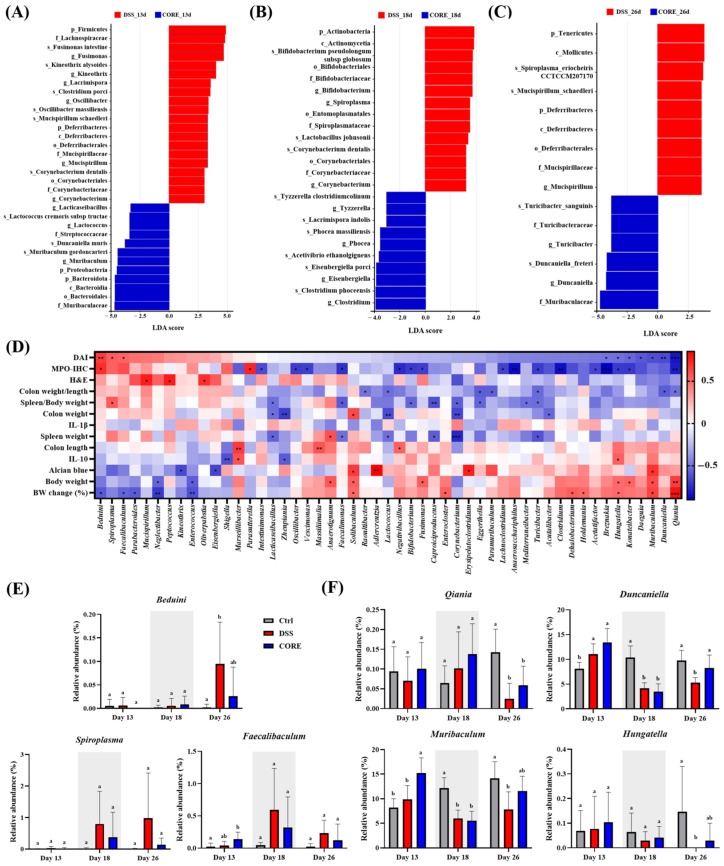
Associations between gut microbial composition and pathological parameters following CORE supplementation in DSS-induced colitis mice. (**A**–**C**) Linear discriminant analysis (LDA) scores of differentially abundant taxa between DSS and CORE groups at days 13, 18, and 26. (**D**) Heatmap of Spearman correlation analysis between the relative abundance of gut microbial genera (day 26) and pathological indices. Color intensity indicates the strength and direction of the correlation (red, positive; blue, negative), and asterisks denote statistical significance. (**E**,**F**) Relative abundance of genera that were decreased or increased following CORE supplementation among groups. Data are presented as mean ± SEM (*n* = 8 per group). Statistical significance was analyzed using one-way ANOVA followed by Tukey’s post hoc test. Different letters (a, b) above the bars indicate statistically significant differences at *p* < 0.05. * *p* < 0.05, ** *p* < 0.01, *** *p* < 0.001.

To further characterize the microbiota-modulatory effects of CORE, differential taxa between experimental groups were identified using LEfSe analysis (LDA > 3.0) at days 13, 18, and 26 (Figure 5A–C), enabling evaluation of temporal changes in gut microbial composition following CORE supplementation. At baseline (day 13), prior to DSS administration, two weeks of CORE pre-intervention increased the relative abundance of *Muribaculaceae*, *Lacticaseibacillus*, *Lactococcus*, and *Muribaculum*, while reducing *Mucispirillum schaedleri* (Figure 5A), a bacterium previously associated with intestinal inflammation [29]. Following DSS administration (day 18), CORE-associated differences became less pronounced, indicating that DSS-induced perturbation may dominate microbial community structure during the acute inflammatory phase. Only limited and variable differences were observed between DSS and CORE groups (Figure 5B). These findings suggest that DSS-induced perturbation may override or obscure CORE-mediated microbiota modulation during the acute inflammatory phase. During the recovery phase (day 26), distinct microbial patterns re-emerged. DSS-treated mice exhibited enrichment of *Spiroplasma eriocheiris* and *Mucispirillum schaedleri*, whereas CORE-treated mice showed higher relative abundances of *Muribaculaceae*, *Duncaniella*, and *Turicibacter* (Figure 5C). Notably, this pattern partially resembled the baseline microbiota profile observed in CORE-treated mice prior to DSS exposure (Figure 5A), suggesting a restoration of CORE-associated microbial features following inflammatory stress. Collectively, these results indicate that DSS-induced colitis markedly reshapes gut microbial composition and may transiently mask CORE-mediated effects during the acute phase, whereas CORE-associated microbiota signatures re-emerge during recovery.

Spearman’s rank correlation analysis was performed to evaluate associations between gut microbial genera and disease-related parameters across samples from DSS-treated mice (DSS and CORE groups). As shown in Figure 5D, a total of 51 genera identified by microbiota analysis were included. These genera comprised taxa that were either enriched or depleted following DSS exposure and CORE treatment. The analysis revealed that the genera of *Qiania*, *Duncaniella*, *Muribaculum*, and *Hungatella* were negatively correlated with colitis severity-related parameters, whereas the genera of *Beduini*, *Spiroplasma*, and *Faecalibaculum* showed positive correlations with disease-associated indices (Figure 5D). To further examine these associations, temporal abundance analysis was performed. Consistent with the correlation patterns, CORE supplementation was associated with decreased relative abundance of *Beduini*, *Spiroplasma*, and *Faecalibaculum*, while increasing the abundance of *Qiania*, *Duncaniella*, *Muribaculum*, and *Hungatella* (Figure 5E,F). Collectively, these findings suggest that CORE supplementation is associated with shifts in microbial taxa that correlate with disease severity in DSS-induced colitis.

## 3. Discussion

Gut diseases, such as inflammatory bowel disease and other gastrointestinal inflammatory disorders, have emerged as a health issue worldwide [30]. Their pathogenesis is often linked to intestinal barrier disruption, immune dysregulation, inflammation activation, and gut dysbiosis [4]. Increasing evidence suggests that modulation of the intestinal microenvironment is a promising strategy for alleviating inflammation and restoring gut homeostasis [2]. In this context, probiotics have emerged as a widely studied and accessible intervention for maintaining intestinal health [31]. In the present study, using a DSS-induced colitis mouse model, we demonstrated that pre-administration of the multi-strain probiotic formulation CORE significantly attenuated colitis severity. CORE supplementation reduced DAI scores and body weight loss, improved distal colonic histopathology, and modulated inflammatory cytokine profiles. Notably, CORE-treated mice exhibited accelerated recovery following DSS-induced injury, suggesting that CORE supports intestinal resilience rather than acting solely as an anti-inflammatory agent.

DSS-induced colitis is characterized by dynamic and coordinated alterations in cytokine networks, reflecting the interplay between innate and adaptive immune responses [32]. In this study, CORE supplementation modulated the expression of PTX2, CHI3L1, CXCL13, and MMP-2, suggesting a multifaceted effect on post-inflammatory recovery through attenuation of epithelial stress, normalization of immune cell recruitment, and reduction of excessive tissue remodeling. PTX2 is an innate pentraxin that binds Fcγ receptors and regulates inflammatory resolution, macrophage polarization, complement activation, and tissue repair processes through pathways such as PI3K/ERK signaling [23,33]. A previous study has shown that circulating PTX2 levels are elevated during intestinal inflammation and correlate with disease severity in experimental colitis [34]. CHI3L1, a glycoprotein of the glycoside hydrolase family 18 that lacks chitinase activity, has been recognized as a marker of persistent mucosal inflammation and epithelial stress [35]. Elevated CHI3L1 has been reported in inflammatory bowel disease and colitis-associated neoplasia, where it contributes to intestinal inflammation and epithelial remodeling through PI3K/Akt/mTOR, MAPK/ERK, STAT3, and Wnt/β-catenin signaling pathways [36,37,38,39]. As shown in Figure 3B, CORE supplementation significantly reduced circulating PTX2 and CHI3L1 levels during the recovery phase, suggesting improved resolution of residual inflammation and attenuation of epithelial stress after DSS-induced injury. Similarly, CXCL13 is a chemokine that is involved in the development of secondary lymphoid organs and the recruitment of adaptive immune cells [24]. Elevated CXCL13 levels have been reported in both patients with inflammatory bowel disease and DSS-induced colitis models, where it is associated with chronic inflammatory signaling and disease severity [40,41]. MMP-2, a key mediator of extracellular matrix turnover and tissue remodeling, degrades basement membrane components and facilitates epithelial restitution, leukocyte migration, and wound repair [42,43]. Notably, CORE supplementation significantly decreased CXCL13 and MMP-2 levels even though the two markers were not significantly elevated in the DSS group alone. This finding suggests that CORE may not only suppress overt inflammatory responses but also modulate immune activation and tissue remodeling processes during post-inflammatory recovery. Together, these results support a multi-layered protective effect of CORE involving suppression of systemic and mucosal inflammatory signaling, restoration of epithelial integrity, and improved post-inflammatory healing. However, because the present study did not directly evaluate the activation status of PI3K/Akt/mTOR, MAPK/ERK, Wnt/β-catenin, and NF-κB signaling pathways, this mechanistic interpretation should be considered preliminary and requires further experimental validation.

Beyond inflammation, modulation of the gut microbiota represents a key mechanism underlying probiotic function [44]. In this study, CORE supplementation influenced microbial composition both prior to and following DSS exposure. Notably, CORE did not fully prevent DSS-induced disruption of microbial diversity or global community structure, particularly during the acute inflammatory phase, where DSS-induced perturbation appeared to dominate microbial dynamics. Instead, CORE exerted a context-dependent effect characterized by attenuation of early diversity loss and re-emergence of specific microbial taxa during recovery. These findings indicate that CORE enhances microbiota resilience, the capacity of the microbial ecosystem to resist perturbation and recover following stress, rather than directly restoring microbial composition. This distinction between microbiota restoration and resilience is important. While many probiotic studies emphasize the ability to restore microbial composition following perturbation, complete restoration is often difficult to achieve under inflammatory conditions. In contrast, enhancing resilience may represent a more biologically relevant and achievable outcome. In the present study, CORE attenuated the early decline in microbial diversity during DSS exposure and facilitated the re-establishment of specific microbial features during recovery, despite persistent alterations in global community structure. These observations suggest that probiotic interventions may exert beneficial effects by stabilizing microbial ecosystems and promoting recovery dynamics, rather than by fully reversing dysbiosis.

Consistent with this interpretation, CORE supplementation increased the abundance of several taxa associated with host-beneficial functions, including members of the *Muribaculaceae* family (e.g., *Duncaniella* and *Muribaculum*) and *Turicibacter*. *Muribaculaceae* are prevalent members of the gut microbiota and have been linked to extended lifespan in mice, with their abundance inversely associated with inflammation and reduced levels implicated in colitis pathogenesis [45]. *Duncaniella* has been suggested to confer protection against DSS-induced colonic injury [45], while *Muribaculum* abundance has been positively correlated with butyrate level [46]. In addition, *Turicibacter*, a common gut commensal, has been implicated in the regulation of host lipid and bile acid metabolism [47], further supporting its potential contribution to intestinal homeostasis. Functional prediction analysis using PICRUSt2 further supported this microbial shift by identifying six CORE-associated metabolic pathways: acetyl-CoA fermentation to butanoate II (PWY-5676), fatty acid β-oxidation I (FAO-PWY), glutaryl-CoA degradation (PWY-5177), inosine-5′-phosphate biosynthesis III (PWY-7234), pyruvate fermentation to butanoate (CENTFERM-PWY), and the superpathway of acidogenic fermentation in *Clostridium acetobutylicum* (PWY-6590) (Figure S3). Notably, several of these pathways are directly involved in short chain fatty acid (SCFA) biosynthesis, particularly butyrate production. Based on these predicted functional changes, we speculate that CORE supplementation may have partially restored SCFA production impaired by DSS treatment, thereby contributing to improved mucosal recovery and maintenance of intestinal homeostasis. Although SCFAs were not directly measured in this study, these findings suggest that CORE may enhance microbial metabolic outputs that support intestinal resilience and post-inflammatory recovery.

Despite these findings, several limitations should be acknowledged. First, direct metabolomic measurements were not performed to confirm changes in SCFA levels, and thus functional inferences remain indirect. Second, potential interactions among strains within the CORE formulation were not investigated, despite the possibility of synergistic or antagonistic effects in multi-strain consortia. Third, mechanistic pathways linking CORE to host signaling networks, such as NF-κB activation or epithelial barrier regulation, were not directly examined. Future studies integrating metabolomics, mechanistic pathway analysis, and strain-level interaction studies will be essential to further elucidate the functional basis of CORE activity.

## 4. Materials and Methods

### 4.1. Probiotics Consortium Preparation

The multi-strain probiotic formulation Neuralli^TM^-CORE (CORE) was provided by Bened Biomedical Co., Ltd. CORE consists of five different bacterial species: *Bifidobacterium longum*, *Lactococcus cremoris*, *Lacticaseibacillus paracasei*, *Lacticaseibacillus rhamnosus*, and *Limosilactobacillus fermentum*. The formulation was prepared by combining lyophilized powder from each strain to achieve an equal concentration of 2 × 10^8^ colony-forming unit (CFU) per strain in the final mixture, with viable bacterial counts determined by CFU enumeration, and administered to mice at a total dose of 1 × 10^9^ CFU per day.

### 4.2. DSS-Induced Colitis Mouse Model

This intervention study was designed to evaluate the effects of the CORE probiotic formulation in a DSS-induced colitis mouse model. Male C57BL/6J mice (7 weeks old, weight rage 20~23 mg) were obtained from the National Center for Biomodels (NCB), National Institutes of Applied Research (NIAR) (Taipei, Taiwan) and acclimatized for one week under controlled conditions (22 ± 1 °C, 55–65% humidity, 12 h light–dark cycle). Mice were provided *ad libitum* access to a standard chow diet and sterilized drinking water. After acclimation, mice were randomly assigned to three groups (*n* = 10 per group): Control (Ctrl), DSS, and CORE. As illustrated in Figure 1A, mice received daily oral gavage of either 200 μL phosphate-buffered saline (PBS) or CORE (1 × 10^9^ CFU in 200 μL PBS) through the entire experimental period. Following two weeks of pre-treatment, colitis was induced in the DSS and CORE groups by administering 2% (*w*/*v*) DSS (M.W. 36–50 kDa; MP Biomedicals, Santa Ana, CA, USA) in the drinking water for 7 days, followed by a recovery period with sterilized water for an additional 7 days. At the end of the experimental period, mice were euthanized by CO_2_ inhalation. Blood samples were centrifuged at 1500× *g* for 10 min, and serum was subsequently collected. Distal colon tissues were harvested, rinsed with PBS, measured for length, and photographed. Fecal samples were collected and stored at −80 °C for subsequent microbiota analysis. All experimental procedures were approved by the Institutional Animal Care and Use Committee (IACUC) of the NCB, NIAR.

### 4.3. Evaluation of Disease Activity Index (DAI)

The disease activity index (DAI) was assessed as previously described [48]. DAI scores were determined based on three parameters: body weight loss, stool consistency, and the presence of fecal blood. Body weight loss was scored as follows: 0, no loss; 1, 0–5%; 2, 5–10%; 3, 10–20%; and 4, >20% relative to baseline body weight on the first day of DSS administration (day 15). Stool consistency was scored as: 0, normal; 2, loose stools; and 4, watery diarrhea. The presence of gross blood in the stool was scored as: 0, negative; and 4, positive. Body weight was recorded every two days throughout the experimental period. The total DAI score was calculated as the sum of the three individual parameters, yielding a composite score ranging from 0 to 12. The assessment was performed in three independent experiments (*n* = 25–30 per group), and data from all experiments were combined for statistical analysis.

### 4.4. Histopathological Analysis

Histological sample preparation and staining were performed by the NCB, NIAR (Taipei, Taiwan). Colon tissues were fixed in 4% paraformaldehyde, embedded in paraffin, and sectioned for histological analysis. Tissue architecture and colonic injury were evaluated using hematoxylin and eosin (H&E) staining. Goblet cells and mucus production were assessed by Alcian blue staining. Myeloperoxidase (MPO) expression was evaluated by immunohistochemistry (IHC) using an anti-MPO primary antibody (1:100, PA5-16672, Invitrogen, Carlsbad, CA, USA). Stained sections were imaged using a light microscope (Olympus CX43, Evident Scientific, Tokyo, Japan), and quantitative analysis was performed using ImageJ software (v1.54d, NIH, Bethesda, MD, USA). Histological lesions were scored based on lesion depth, severity of inflammation (including mucosal damage and inflammatory cell infiltration), and the presence of lymphoid hyperplasia, crypt ectasia, edema, erosion, and ulceration [49].

### 4.5. Measurement of Inflammatory Cytokines

Cytokine profiling was performed using a commercial proteome array kit (Proteome Profiler Mouse Cytokine Array Kit, Catalog #ARY028, R&D Systems, Minneapolis, MN, USA) according to the manufacturer’s instructions. Briefly, array membranes were blocked for 1 h, incubated with serum samples overnight at 4 °C, washed, and subsequently incubated with a detection antibody cocktail followed by streptavidin–horseradish peroxidase (HRP) for 30 min at room temperature. Signals were visualized using a chemiluminescence imaging system (Invitrogen iBright FL1500). Cytokine array analysis was performed using samples from three independent experiments. Each sample was analyzed in technical duplicates, and the average of duplicate measurements was used as a single biological replicate for statistical analysis. The levels of IL-1β and IL-10 in distal colon tissues were quantified using enzyme-linked immunosorbent assay (ELISA) kits (IL-1β: 88-7013-88; IL-10: 88-7105-88, Invitrogen, USA), according to the manufacturer’s instructions.

### 4.6. Full-Length 16S rRNA Gene Amplicons Generation and Sequencing

Full-length 16S rRNA gene amplification, library preparation, and sequencing were performed following the PacBio^®^ official protocol (Procedure Checklist: Amplification of Full-Length 16S Gene with Barcoded Primers for Multiplexed SMRTbell Library Preparation and Sequencing). The V1–V9 region of the bacterial 16S rRNA gene was amplified using KAPA HiFi HotStart ReadyMix (Roche, South San Francisco, CA, USA) under the recommended PCR conditions. Barcoded universal primers were used for amplification with the following sequences: forward primer, 5′-Phos/GCATC-[16-base barcode]-AGRGTTYGATYMTGGCTCAG-3′; reverse primer, 5′-Phos/GCATC-[16-base barcode]-RGYTACCTTGTTACGACTT-3′. PCR products were verified by 1% agarose gel electrophoresis, and amplicons of approximately 1500 bp were purified using AMPure PB beads (Pacific Biosciences, Menlo Park, CA, USA). Purified amplicons were pooled in equimolar concentrations, and 500–1000 ng of DNA was subjected to DNA damage repair, followed by end-repair, A-tailing, and ligation of SMRTbell hairpin adapters. Following purification to remove adapter dimers, SMRTbell libraries were prepared using the Sequel II Primer 3.1 and Sequel II Binding Kit 3.1 for primer annealing and polymerase binding. Sequencing was performed on a PacBio^®^ Sequel IIe platform in circular consensus sequencing (CCS) mode to generate HiFi reads with a predicted accuracy of ≥Q30 (Phred scale).

### 4.7. Bioinformatic Analysis for Full-Length 16S rRNA Metagenomics

Raw HiFi reads were processed using the DADA2 pipeline (v1.20) for quality filtering, dereplication, error model learning, and chimera removal to generate amplicon sequence variants (ASVs) with single-nucleotide resolution (Figure S4A,C,E). Taxonomic assignment of representative ASV sequences was performed using QIIME2 (v2022.11; https://qiime2.org/) against the NCBI reference database (January 2023 release). To account for differences in sequencing depth across samples, ASV abundance data were rarefied to the minimum sequencing depth using the QIIME script single_rarefaction.py (Figure S4B,D,F). α-diversity indices, including Shannon, Simpson, and Pielou indexes, and beta diversity based on principal coordinates analysis (PCoA) were calculated using R software (v4.0.5) based on the rarefied dataset. Data visualization and statistical analyses were performed using the microeco (v2.2.0), plotly (v6.7.0), and ggplot2 (v3.4.2) R packages. Differentially abundant taxa were identified using linear discriminant analysis effect size (LEfSe).

### 4.8. Functional Prediction of Gut Microbiota Using PICRUSt2

Functional prediction of microbial communities was performed using Phylogenetic Investigation of Communities by Reconstruction of Unobserved States 2 (PICRUSt2). ASVs generated from 16S rRNA gene sequencing were used as input and processed through the PICRUSt2 pipeline, including sequence placement, hidden-state prediction, and metagenome inference. Predicted gene family abundances were annotated against the Kyoto Encyclopedia of Genes and Genomes (KEGG) database, and pathway-level functional profiles were inferred using MetaCyc pathway annotations. Differential pathway abundance among groups was analyzed using R software (v4.0.5).

### 4.9. Statistical Analysis

Data were analyzed using GraphPad Prism (version 10) and are presented as mean ± standard error of the mean (SEM). Normality of data distribution was assessed using the Shapiro–Wilk test, and homogeneity of variances was evaluated using the Brown–Forsythe test. Differences among multiple groups were analyzed using one-way analysis of variance (ANOVA) followed by Tukey’s post hoc test when assumptions of normality and equal variance were satisfied. When variance homogeneity was not met, Welch’s ANOVA was applied, followed by Dunnett’s T3 multiple comparisons test. For pairwise comparisons, Welch’s *t*-test was used when data was not normally distributed. Spearman’s rank correlation analysis was performed to evaluate associations between gut microbial genera and pathological parameters across samples. To assess temporal changes in microbial diversity, within-individual changes in Shannon diversity between day 13 and day 18 (ΔShannon) were calculated as the difference between the two time points. Differences in ΔShannon between groups were evaluated using an unpaired two-tailed Welch’s *t*-test. Longitudinal changes in α-diversity across time points (days 13, 18, and 26) were analyzed using two-way ANOVA with group and time as fixed factors, including their interaction term. When appropriate, post hoc comparisons were performed using Tukey’s test. Temporal trends were visualized using trajectory plots based on group mean values at each time point. *p* value < 0.05 was considered statistically significant.

## 5. Conclusions

In this study, the newly developed multi-strain probiotic formulation CORE effectively attenuated DSS-induced increases in disease activity index and body weight loss, protected distal colonic tissue integrity, and modulated inflammatory cytokine responses. Notably, CORE did not fully restore gut microbiota composition but instead enhanced microbiota resilience, as evidenced by attenuation of early diversity loss and re-emergence of specific microbial taxa during recovery. Collectively, these findings suggest that CORE alleviates intestinal inflammation and supports recovery by stabilizing the gut microbial ecosystem under inflammatory stress, highlighting its potential as a multi-strain probiotic strategy for maintaining intestinal homeostasis.

## Data Availability

The original contributions presented in this study are included in the article/Supplementary Materials. Further inquiries can be directed to the corresponding authors.

## Supplementary Materials

The following supporting information can be downloaded at: https://www.mdpi.com/article/10.3390/ijms27115108/s1.
