# Supplementary material for: A Probiotic Mixture Neuralli™-CORE Attenuates DSS-Induced Colitis by Enhancing Gut Microbiota Resilience in Mice
Source: Int J Mol Sci. 2026 Jun 4;27(11):5108. doi: 10.3390/ijms27115108 (PMC13256931; doi:10.3390/ijms27115108)
Supplement: Supplementary file 1 [file ijms-27-05108-s001.zip › ijms-4316097-supplementary.pptx]

## Slide 1
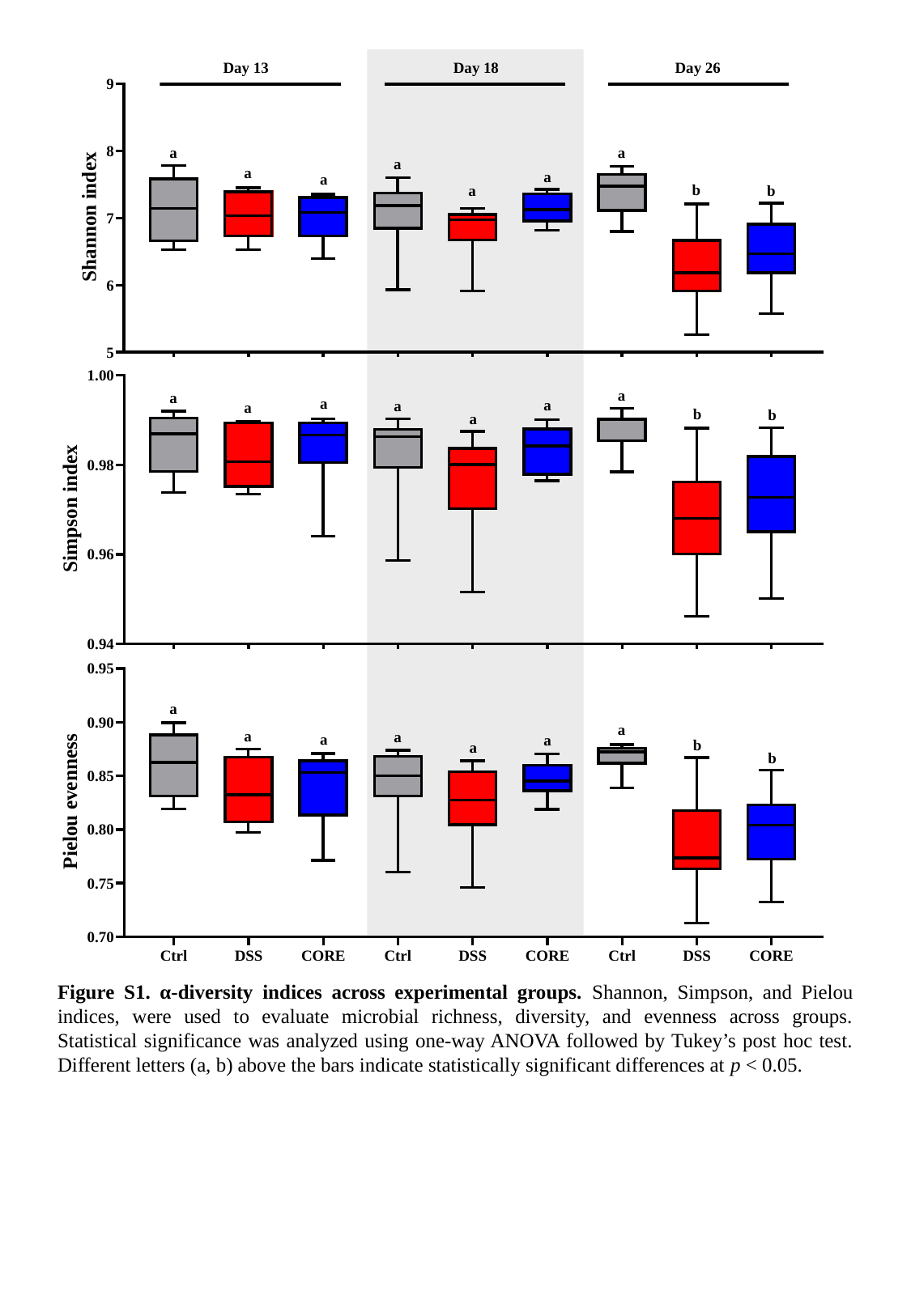

Figure S1. α-diversity indices across experimental groups. Shannon, Simpson, and Pielou indices, were used to evaluate microbial richness, diversity, and evenness across groups. Statistical significance was analyzed using one-way ANOVA followed by Tukey’s post hoc test. Different letters (a, b) above the bars indicate statistically significant differences at p < 0.05.

## Slide 2
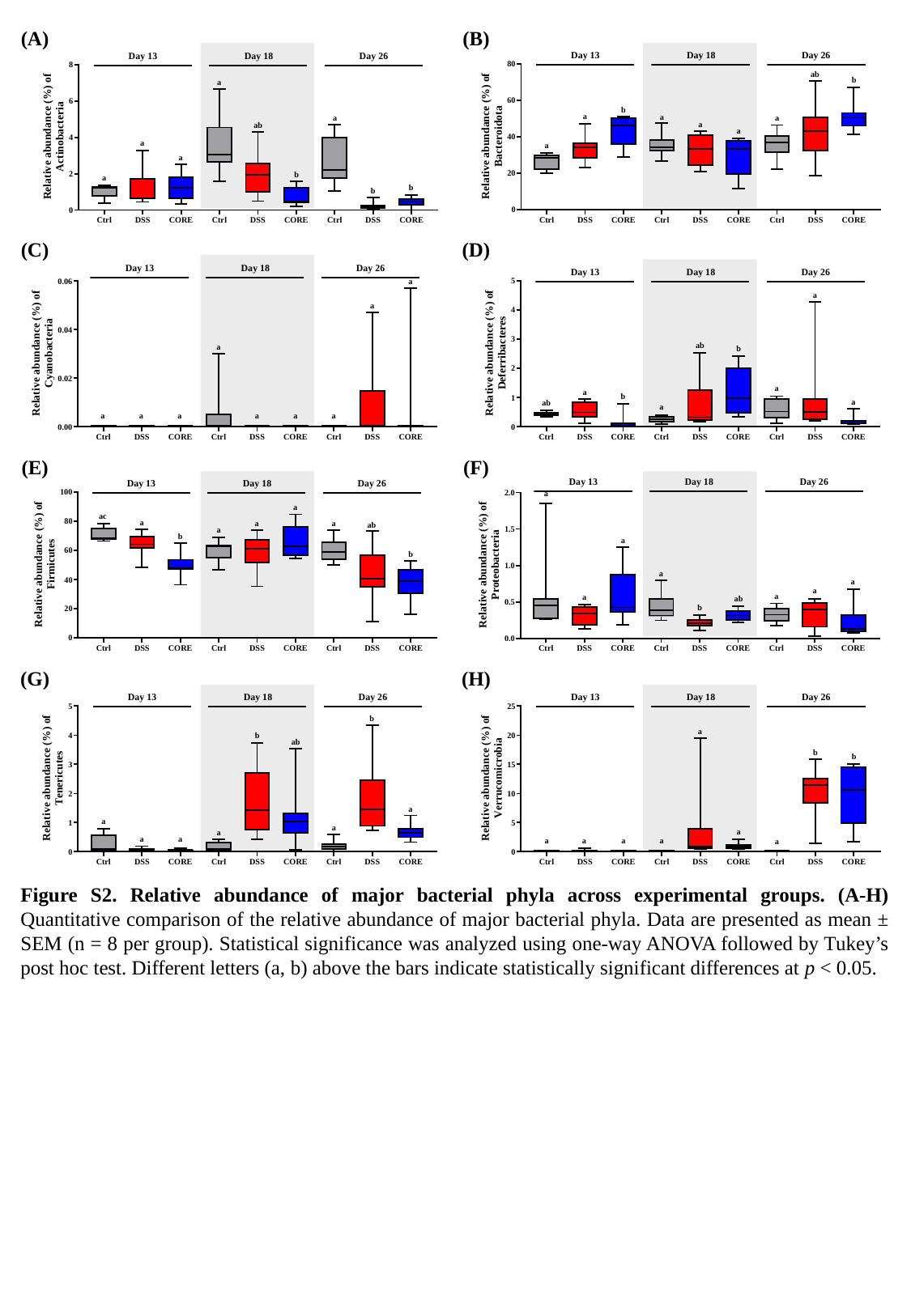

(A)
(B)
(C)
(D)
(E)
(F)
(G)
(H)
Figure S2. Relative abundance of major bacterial phyla across experimental groups. (A-H) Quantitative comparison of the relative abundance of major bacterial phyla. Data are presented as mean ± SEM (n = 8 per group). Statistical significance was analyzed using one-way ANOVA followed by Tukey’s post hoc test. Different letters (a, b) above the bars indicate statistically significant differences at p < 0.05.

## Slide 3
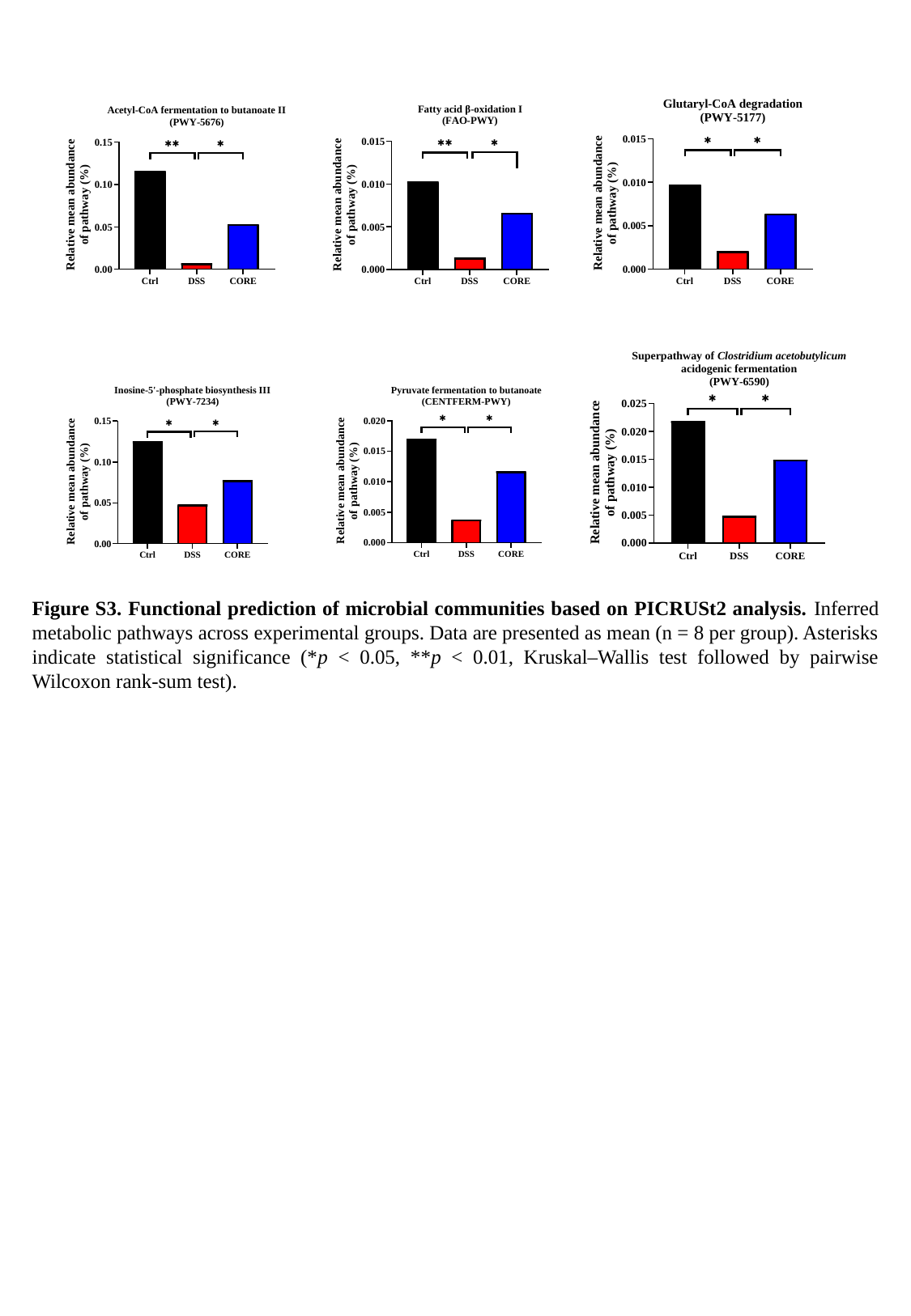

Figure S3. Functional prediction of microbial communities based on PICRUSt2 analysis. Inferred metabolic pathways across experimental groups. Data are presented as mean (n = 8 per group). Asterisks indicate statistical significance (*p < 0.05, **p < 0.01, Kruskal–Wallis test followed by pairwise Wilcoxon rank-sum test).

## Slide 4
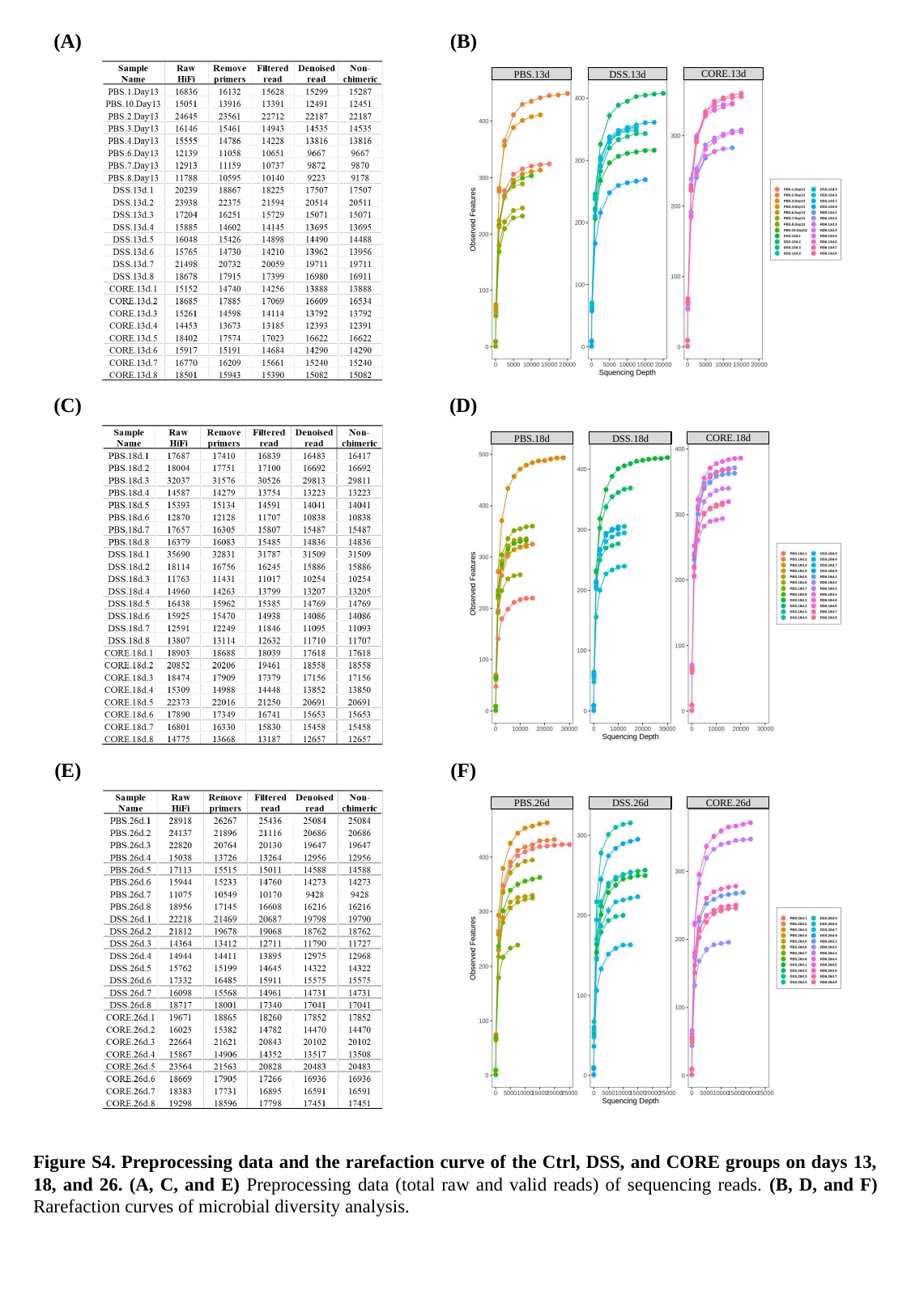

(A)
(B)
CORE.13d
PBS.13d
DSS.13d
(C)
(D)
CORE.18d
PBS.18d
DSS.18d
(E)
(F)
CORE.26d
PBS.26d
DSS.26d
Figure S4. Preprocessing data and the rarefaction curve of the Ctrl, DSS, and CORE groups on days 13, 18, and 26. (A, C, and E) Preprocessing data (total raw and valid reads) of sequencing reads. (B, D, and F) Rarefaction curves of microbial diversity analysis.
